# The Utility of BDNF Detection in Assessing Severity of Huntington’s Disease

**DOI:** 10.3390/jcm10215181

**Published:** 2021-11-05

**Authors:** Klaudia Plinta, Andrzej Plewka, Krzysztof Pawlicki, Nikola Zmarzły, Magdalena Wójcik-Pędziwiatr, Marcin Rudziński, Agnieszka Krzak-Kubica, Magdalena Doręgowska-Stachera, Monika Rudzińska-Bar

**Affiliations:** 1 Neurology Department, Institute of Medical Science, University of Opole, 45-040 Opole, Poland; klaudia_plinta@o2.pl; 2 Institute of Health Sciences, University of Opole, 45-040 Opole, Poland; aplewka@sum.edu.pl; 3 Faculty of Medical Sciences, Medical University of Silesia, 40-055 Katowice, Poland; pawlicki@sum.edu.pl; 4 Department of Histology, Cytophysiology and Embryology, Faculty of Medicine, University of Technology in Katowice, 41-800 Zabrze, Poland; 5 Department of Neurology, Faculty of Medicine and Health Sciences, Andrzej Frycz Modrzewski Krakow University, 30-705 Krakow, Poland; magdalena.wojcik4@wp.pl (M.W.-P.); mdoregowska@afm.edu.pl (M.D.-S.); mrudzinska@afm.edu.pl (M.R.-B.); 6 Department of Otolaryngology, Jagiellonian University Medical College, The University Hospital in Krakow, 30-688 Krakow, Poland; mtrudzin@gmail.com; 7 Vito-Med Hospital in Gliwice, 44-100 Gliwice, Poland; agukrzak@gmail.com

**Keywords:** Huntington’s disease, BDNF, disease severity

## Abstract

Brain-derived neurotrophic factor (BDNF) is involved in the survival and maturation of neurons, and also promotes and controls neurogenesis. Its levels are lowered in many neurodegenerative diseases, including Huntington’s disease (HD). Clinical pictures of HD can be very diverse, which makes it difficult to assess its severity; however, molecular markers may be helpful. The aim of the study was to determine the relationship between HD severity and the plasma BDNF concentration in HD patients. The study recruited 42 patients with diagnosed and genetically confirmed HD and 40 healthy volunteers. BDNF levels were determined in plasma with the enzyme-linked immunosorbent assay (ELISA). Correlations between BDNF levels and clinical profiles and HD severity were also investigated. The BDNF level was significantly lower in HD patients compared to the control. There was no correlation between the BDNF level and motor symptoms and cognitive impairment. In the early disease stages, BDNF levels were associated with a better neurological examination, independence, and functional evaluation, in contrast to later HD stages, where the correlations were inverse. Multidirectional correlations between parameters of saccadic disorders and the BDNF level do not allow for drawing a conclusion, whether or not there is a relationship between the severity of saccadic disorders and the BDNF concentration.

## 1. Introduction

Brain-derived neurotrophic factor (BDNF) is responsible for the growth and development of nerve cells, and determines the survival of striatal neurons, regulates learning and memory, and influences mood and behavior [1,2]. It occurs in all structures of the central nervous system, but its highest concentration was recorded in the hippocampus and cerebral cortex [3]. BDNF is transported to the striatum, where it modulates both the communication between nerve cells and glutamate metabolism, and also has a neuroprotective effect [4]. BDNF is also found in the peripheral nervous system and other tissues of the body. Moreover, BDNF levels are approximately one hundred times higher in serum than in plasma, due to the release of growth factors by platelets [5]. Additional peripheral sources of brain-derived neurotrophic factor are macrophages, lymphocytes, endothelial cells, and smooth muscles. It crosses the blood–brain barrier bidirectionally, and, therefore, the peripheral concentration of this factor may also depend on its production by neurons and glial cells [6,7].

Huntington disease (HD) is a progressive neurodegenerative disorder with a worldwide incidence of 2.71 per 100,000 people [8]. It is caused by a mutation in huntingtin, which disrupts many cellular processes, including the transcription of the BDNF gene, transport of the finished protein, and its interaction with the Tr-kB receptor [9]. In addition, the presence of aggregates of abnormal huntingtin is responsible for further lowering BDNF levels, while, in healthy people, the correct form of huntingtin stimulates the cortical production of BDNF [10,11].

A postmortem study of HD patients showed a reduction in BDNF expression in the caudate and putamen. On the other hand, there was no change in its level in the parietal cortex, temporal cortex, and hippocampus. The region-dependent loss of BDNF expression in the brain of HD patients is consistent with the areas most affected by disease [12]. BDNF levels have also been assessed in the blood, and a decrease in its serum levels has been reported in patients with symptomatic HD [13,14]. There was also a negative correlation with the severity of motor disorders measured with the Unified Huntington’s Disease Rating Scale (UHDRS). Lower BDNF levels coexisted with a greater number of cytosine-adenine-guanine (CAG) repeats and a faster onset of the symptomatic phase of the disease [13,14]. In one cohort, plasma BDNF values increased with disease severity, but these results were not statistically significant. On the other hand, the level of serum BDNF and BDNF mRNA were similar in the study and control groups. There was no correlation between the BDNF level and the number of CAG repeats or the UHDRS. In addition, there were no differences in BDNF levels depending on age or gender, both in HD patients and healthy people, which was contrary to previous reports [15].

The clinical picture of HD can be very diverse, which makes it difficult to precisely assess disease severity. Therefore, supplementing currently used assessment and test scales with molecular markers may be promising. The aim of the study was to determine the relationship between the severity of HD and BDNF plasma concentration in HD patients.

## 2. Materials and Methods

A total of 82 people were recruited for the study, including 42 patients (24 women and 18 men) with diagnosed and genetically confirmed HD and 40 healthy volunteers (20 women and 20 men) without a family history of HD or other neurodegenerative diseases. In addition, the exclusion criteria included hypothyroidism or hyperthyroidism, metabolic diseases, autoimmune diseases, cancer, and acute infectious disease. The patients were treated at the Department of Neurology of the University Clinical Center of the Medical University of Silesia in Katowice. Written informed consent was obtained from all study participants.

### 2.1. Classification and Neurological Assessments in HD Patients

All HD patients underwent a neurological examination with the motor disorder assessment based on the Unified Huntington’s Disease Rating Scale (UHDRS). The following parameters were evaluated: pursuit and rapid eye movements, speech disorders, dystonia and chorea, bradykinesia, and impaired postural reflexes. A total of 0 to 4 points were given for each domain (0—no deviations from normal state, 4—severe disorder) and maximum of 124 points could be obtained. Symptomatic HD phase was diagnosed in patients who scored six or more points.

Based on the total score obtained in the UHDRS motor domain, patients were divided into following groups according to the HD stage: preclinical (0 points), very early (1–13 points), early (14–37 points), intermediate (38–67 points), and advanced (>67 points). Independence in social and life functions, evaluated with a scale ranging from 10% (a patient requiring help from other people in all life activities) to 100% (an independent patient in domestic and social activities), was also used to determine HD severity.

UHDRS scale has proven to be useful, both in the analysis of interdependencies between its individual domains and result repeatability when assessing the same patient by different investigators [16]. Division of patient groups based on the UHDRS motor scale has already been used in previous studies [17,18].

### 2.2. Examination of Cognitive Functions and Mood Disorders in HD Patients

Cognitive functions were assessed using screening tests, such as Mini-Mental State Examination (MMSE), Montreal Cognitive Assessment (MoCA), and Clock Drawing Test (CDT), as well as detailed tests, including Symbol Digital Modality Test (SDMT), verbal fluency (VF), and phonemic fluency (Total Fluency, TF) tests, Trail Making Test (TMT) parts one and two, and Stroop test (ST) parts one, two, and three. A detailed description of performed tests is included in our previously published article [19].

### 2.3. BDNF Protein Level Assessment

Approximately 5 mL of venous blood was collected from all participants into test tubes containing EDTA (Sarstedt, Nümbrecht, Germany). Samples were left at room temperature until the plasma was separated (15 min), and then centrifuged for 10 min at 1000× *g*. The obtained plasma was pipetted into Eppendorf tubes and stored at −70 °C. Plasma BDNF levels were determined with professional ELISA kit (cat. no. SEA011Hu; Cloud-Clone Corp., Houston, TX, USA), according to the manufacturer’s instructions.

### 2.4. Statistical Analysis

Statistical analysis was performed using Statistica 13.1 (StatSoft, Kraków, Poland). The distribution of the data was determined with Kolmogorov–Smirnov test. Mann–Whitney U test or Kruskal–Wallis test with post-hoc Dunn analysis were used for two and three groups, respectively. Correlations were assessed with the Spearman’s test.

## 3. Results

The characteristics of the study group in which plasma BDNF levels were assessed are summarized in Table 1.

In the case of the control group, the mean age of healthy volunteers was 45.94 years (SD: ± 10.5; range: 23–67 years). HD patients were then further classified: 3 as preclinical, 13 to the early disease stage, 21 to the intermediate stage, and 5 to the advanced stage. The duration of the disease was 5.84 years in the early group, 9.04 years in the intermediate group, and 11 years in the advanced group.

The BDNF level was not affected by sex, age, disease duration, number of CAG repeats, or disease severity in HD patients (*p* > 0.05). Table 2 shows plasma levels of BDNF in the study group divided into HD stages and the control group.

**Figure 1:**
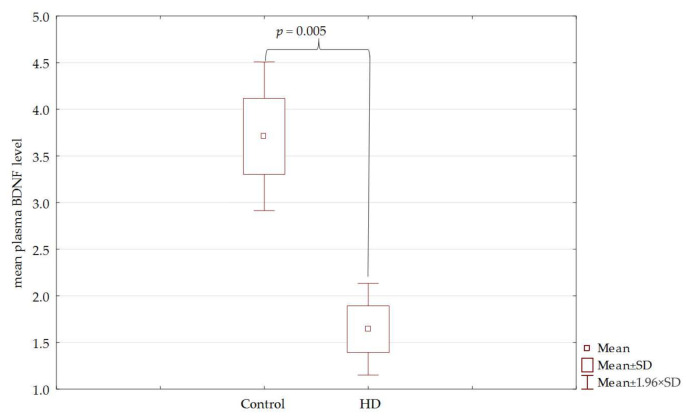
Plasma levels of BDNF in control and patients with HD.

**Figure 2:**
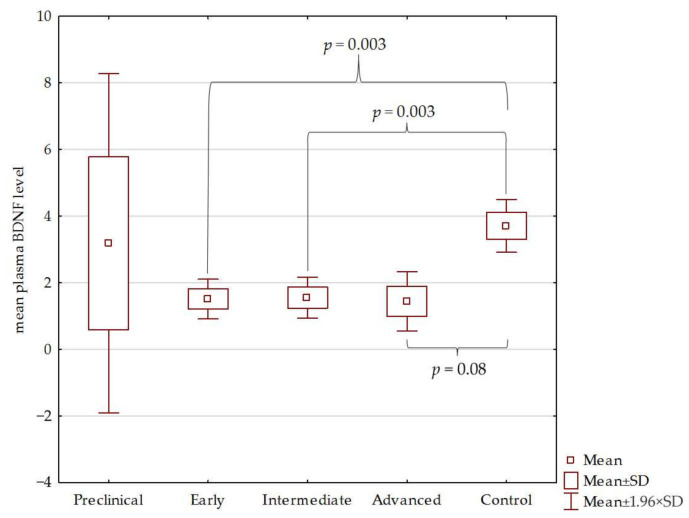
Plasma levels of BDNF in control group and individual HD stages.

Plasma BDNF levels in HD patients were lower than in the control. Similarly, a statistically significant difference in the BDNF level was observed in patients from the early and intermediate stages compared to the control group (*p* < 0.01). On the other hand, the BDNF level in the advanced HD stage was insignificant compared to the control (*p* = 0.08) and preclinical stage (*p* = 0.1). No statistically significant differences were found between plasma levels of BDNF in the preclinical stage compared to the control. The results are shown in Figure 1 and Figure 2.

The largest number of correlations between the BDNF level and elements of neurological examination in HD patients was observed in the advanced group. Single correlations were shown in the early stage, whereas no significant correlations were found in the intermediate stage (Table 3).

In early HD, higher plasma levels of BDNF were found in patients with less severe bradykinesia (*p* = 0.04, R = −0.49), more severe dystonia of the trunk (*p* = 0.04, R = 0.51), and lower limb dystonia (*p* = 0.02, R = 0.62).

In the advanced disease stage, no correlation was found between the BDNF level and bradykinesia. However, correlations between a higher BDNF concentration and lower severity of upper limb dystonia (*p* < 0.01, R = −0.97) and lower limb dystonia (*p* < 0.01, R = −0.97) were revealed. There was also a relationship between high plasma BDNF levels and a higher intensity of ocular motor disorders, both in horizontal and vertical pursuits (*p* = 0.01, R = 0.94), speed of horizontal saccades (*p* = 0.01, R = 0.94), and severity of speech disorders (*p* = 0.04, R = 0.89). The total score on the motor domain of UHDRS in the advanced group was inversely correlated with the BDNF level (*p* = 0.04, R = −0.85), i.e., its lower concentrations occurred in patients with more severe motor symptoms.

In terms of the neuropsychological assessment with MMSE, clock drawing test, MoCA, and Beck’s scale, no correlation was found between the BDNF concentration and the results of these scales at various stages of the disease (Table 4).

The BDNF level correlated with the independence scale (*p* < 0.01, R = 0.69) and functional assessment (*p* = 0.03 R = 0.58); however, only in the early HD stage, where its higher levels were associated with a better performance of patients.

The relationships between the BDNF level and the results of detailed neuropsychological tests are presented in Table 5.

It was observed that a higher plasma BDNF level correlated with both a greater number of correct answers in the Stroop color naming test (*p* = 0.04, R = 0.53) and in the Stroop interference test (*p* = 0.04, R = 0.56), and a shorter trail making test time (*p* = 0.04 R = −0.53) in patients in the early disease stage. Similarly, a higher BDNF level corresponded to better test results in patients in the intermediate group. Patients in the intermediate stage with higher BDNF levels obtained a greater number of incorrect answers corrected in the Stroop word reading test (*p* = 0.04, R = 0.41) and had fewer incorrect selections in the trail making test (*p* = 0.04, R = −0.4). In the intermediate stage, a higher BDNF level was found in patients who had a smaller number of perseverations in the verbal fluency test (*p* = 0.04, R = −0.41).

In the advanced group, a higher BDNF concentration was associated with a lower number of correct answers in the phonemic fluency test (*p* = 0.04, R = −0.86) and in the Stroop word reading test (*p* = 0.04, R = −0.87), and a smaller number of correct selections in the trail making test (*p* = 0.04, R = −0.94).

When analyzing the frequency of the use of drugs affecting the central nervous system (CNS), it was noticed that, in the preclinical group, all patients were taking selective serotonin reuptake inhibitors (SSRIs), such as sertraline and paroxetine, and the tetracyclic antidepressant, mianserin. In the early group, patients also took SSRIs (sertraline, venlafaxine, escitalopram) and neuroleptics (haloperidol, quetiapine, tetrabenazine, risperidone), and one of the patients used hydroxyzine. In the intermediate group, all patients took neuroleptics (olanzapine, tetrabenazine, quetiapine, sulpiride, haloperidol, chlorprothixene), eight patients took SSRIs (sertraline, escitalopram, venlafaxine), and one patient used donepezil. In the group of advanced patients, the highest intake of centrally acting drugs from various groups was observed, i.e., neuroleptics (haloperidol, tetrabenazine, risperidone, promazine), benzodiazepines (clonazepam, lorazepam), mood stabilizers (valproic acid), escitalopram in one patient, and hydroxyzine in one patient.

In summary, the plasma concentration of BDNF in HD patients was significantly lower than in the control group. In the early disease stage, individual tests showed correlations between higher BDNF levels and less severe bradykinesia, better scores in the Stroop test and TMT, and a better functional assessment. There was no evidence of the relationship between the BDNF level and severity of cognitive impairment assessed by MMSE, MoCA, and CDT. However, in the intermediate and advanced stages of HD, higher BDNF levels were found in patients with more severe motor and cognitive symptoms.

## 4. Discussion

Brain-derived neurotrophic factor supports the survival and maturation of neurons in the nervous system and promotes and controls neurogenesis. Moreover, BDNF also has a neuroprotective effect. Studies show that its levels are decreased in many neurodegenerative diseases, including Huntington’s disease [20]. Interestingly, there was a study where the BDNF level increased with HD severity, but observed changes were not statistically significant [15].

In our study, the mean plasma BDNF level in patients (both in all and depending on the stage) was lower than in the control group, which was consistent with previous reports. The highest plasma level of BDNF was recorded in the preclinical group, whereas the lowest was recorded in the intermediate group. Similar results were obtained in the study by Ciammola et al., where the serum BDNF level was significantly lower in HD patients compared to the control. The BDNF level also correlated with the number of CAG repeats and duration of the disease, which was not reported in our study. There were no differences in BDNF levels depending on sex, which was consistent with our observations [14].

In the study by Zucatto et al., BDNF levels in both plasma and serum were assessed in several cohorts of HD patients. In the first cohort, the plasma levels of BDNF were determined in fasting patients, and blood was centrifuged 4 h after collection. The cytokine level was statistically significantly higher in the early, intermediate, and advanced HD groups than in the control. In the second cohort, blood was collected from non-fasting patients, and was centrifuged 2 h after collection. The mean BDNF level was lower than in the first cohort. The BDNF level in patients and the control was comparable. There was a decrease in the plasma BDNF concentration during the transition to the symptomatic stage of the disease. There was no correlation between the BDFN level and the UHDRS, disease stage, number of CAG repeats, and antidepressant treatment, and there were no significant differences in serum BDNF level of HD patients, both in the preclinical and symptomatic stages, compared to the control. Interestingly, in the repeated examination of the same samples, discrepancies in the results were obtained. The authors emphasized the necessity of strict adherence to the study protocol due to the high lability of the determined factor [13]. In our study, we tried to minimize the influence of factors disturbing the measurement through a research plan constructed in the methodology.

Our study showed a correlation between a low level of plasma BDNF and greater severity of bradykinesia, but not dystonia of the trunk and lower limb in the early disease stage. However, in the advanced stage of the disease, correlations between higher plasma BDNF levels and lower severity of upper limb and lower limb dystonia have been reported. A higher BDNF level was associated with a severity of eye movement disorders, both in the direction of eye pursuit and speed of horizontal saccades, as well as a severity of speech disorders. The total score on the motor UHDRS in the advanced group showed that lower BDNF concentrations occurred in patients with more severe motor symptoms. Analyzing our results, it can be assumed that, in the advanced HD stage, an increased level of BDNF is associated with the intensification of oculomotor and dysarthria disorders, but not with dystonic disorders, which are characteristic of the late stages of the disease. A positive correlation between the increase in the BDNF level and features of dystonia was evident in the early HD stage. In the advanced stage, the correlation turned negative, i.e., a lower BDNF concentration was more often associated with dystonic disorders. Perhaps BDNF has a protective effect on the development of neurological symptoms in the early stage (patients with higher BDNF levels showed less slowness of movement), and, in the late stage, a higher BDNF correlated with lower dystonia severity. However, in the late stage of HD, it is no longer possible to compensate for oculomotor and dysarthria disorders, or, paradoxically, the mechanism that reduces the severity of dystonia indirectly negatively affects eye movement and speech disorders. The plasma levels of BDNF between the early and advanced stages were not statistically significantly different, and, therefore, its unchanged amount most likely determines the different effect on the clinical picture.

In Huntington’s disease, the striatum is selectively damaged. This mainly concerns the early and intermediate stages of the disease, whereas global cerebral atrophy is reported in patients in advanced stages. BDNF is a regulator of the nerve cell survival/apoptosis cycle. One of the modulators of BDNF activity in the CNS is huntingtin. In the case of its mutant form, BDNF expression is reduced. In a study by Canals et al., it was shown that an increased number of CAG repeats and a high level of expression of mutant huntingtin in cell cultures correlate with a decrease in BDNF levels [21]. Aggregates of the mutant form of huntingtin are responsible for this decline. In the same study, the authors, using animal models, showed that the reduction in the endogenous BDNF level coexists with the earlier onset of motor symptoms and their severity. The decreased level of BDNF mRNA measured in the cerebral cortex correlated with the severity of striatal atrophy, but was not associated with the disappearance of neurons of the cerebral cortex itself. This cytokine is transported to the striatum from the cerebral cortex, and, thus, its decreased expression may prevent it from being adequately abundant in striatum [21]. This result is consistent with previous reports on both animal models and human studies by Zuccato et al. [9]. In addition, this thesis is supported by the study by Xie et al. [22] and Giralt et al. [23], in which, in animal models of HD, it was shown that increasing cortical BDNF levels was associated with less atrophy of striatal neurons, lower severity of motor disorders, and better cognitive processes. Therefore, BDNF seems to be a worthy marker that indirectly assesses the severity of neurodegeneration in HD. This is particularly important because the current reports, e.g., study by Pillai et al., have shown that the degree of severity of neurodegeneration found in autopsy examinations does not correlate with the results of cognitive tests, also included in the UHDRS assessment. The study showed that, in the group of patients who underwent MMSE tests before death, the results of the tests assessing cognitive abilities were similar between the groups of advanced and intermediate changes found in the autopsy [24].

When assessing detailed cognitive functions, it was noticed that, in the early stages of the disease, high BDNF levels were associated with better test results. Interestingly, in the advanced group, no correlation between higher BDNF concentrations and better results of detailed cognitive tests was observed. This may further indicate a potentially protective effect of BDNF on the performance of patients and their cognitive functions in the early disease stages, which is exhausted in the advanced stage.

Gutierrez et al. reported that BDNF is gaining more and more attention as a potential HD marker, but conflicting results make it difficult to judge its usefulness [25]. In turn, Grah et al., showed a significant decrease in serum BDNF levels in HD patients with suicide attempts, suggesting that this cytokine could be a prognostic marker for suicidal behavior [26]. Results obtained in many laboratories show that the BDNF level decreases during the ongoing neurodegenerative disease. In addition to assessing its usefulness as a biomarker, its therapeutic potential is also studied. Zimmermann et al. transplanted embryonic stem cell-derived neural progenitors overexpressing BDNF into a HD mouse model, which promoted regeneration and recovery [27]. Zuccato et al. showed that, during disease, the supply of cortical BDNF to the striatum is strongly reduced, which may lead to striatal sensitization [10]. The delivery of BDNF may therefore be a useful treatment strategy for Huntington’s disease.

The assessment of the BDNF level is burdened with an error related to the influence of drugs and treatments. Antidepressants and, though rarely used in HD, memantine, riluzole, or cystamine, have a modulating effect on the BDNF level. The use of the selective serotonin reuptake inhibitor to increase the central production of BDNF is debatable due to the inconclusive results of studies conducted so far. From the group of antidepressants, parokesitin, fluoxetine, and lithium salts have been mentioned as potentially increasing BDNF levels [28].

The analysis of used drugs reflects the natural course of Huntington’s disease, in which, depressive mood disorders dominate in the initial stages of the disease, including the pre-motor phase. Psychotic disorders requiring the administration of neuroleptic drugs are more frequently observed in symptomatic patients. Patients in advanced stages of HD often require psychiatric therapy, hence the intake of valproic acid (in doses corresponding to the mood-stabilizing effect) and sedative drugs from the benzodiazepine group that are used mainly at night.

Our study did not exclude patients taking symptomatic drugs in HD, whose withdrawal was impossible, which could have influenced the obtained results. It is therefore important to consider potential drug–protein interactions when drawing conclusions. Moreover, the main limitation of our study was the relatively small study group. Despite the aforementioned limitations, BDNF seems to be a potential diagnostic marker, but has limited promise as a progression marker, which is in line with previous reports.

## 5. Conclusions

Significantly lower levels of plasma BDNF were recorded in HD patients compared to the control, which is consistent with previous reports. Taking into account disease severity, a significant reduction in the BDNF value was noted in the early and intermediate stages. There was no correlation between the BDNF level and motor symptoms and cognitive impairment. Higher levels of BDNF in the early stages of the disease were associated with a better neurological examination, independence, functional evaluation, and an individual cognitive function assessment. In contrast, in the later stages of HD, the correlations were inverse. Multidirectional correlations between parameters of saccadic disorders and the BDNF level do not allow for drawing a conclusion, whether or not there is a relationship between the severity of saccadic disorders and the BDNF concentration.

## Figures and Tables

**Table 1:** Characteristics of the study group of HD patients in whom plasma BDNF levels were determined.

Variables	$\bar{X\;}\pm \;\mathrm{SD}$	Range
Age	49.95 ± 12.38	22–70
CAG repeats	44.18 ± 4.89	40–63
Motor UHDRS	43.78 ± 21.31	0–80
Years of education	13.92 ± 3.65	8–27

$\bar{X\;}$ ± SD—mean ± standard deviation, UHDRS—Unified Huntington Disease Rating Scale, CAG—cytosine-adenine-guanine.

**Table 2:** Plasma levels of BDNF in study group divided into HD stages and control group.

HD	BDNF Level $\bar{X\;}\pm \;\mathrm{SD}$	Range
Preclinical	3.18 ± 4.49	0–8.33
Early	1.5 ± 1.09	0–3.6
Intermediate	1.55 ± 1.43	0–1.43
Advanced	1.44 ± 1.01	0–1.01
All HD patients	1.64 ± 1.62	0–8.33
Control group	3.71 ± 2.53	0–10.27

$\bar{X\;}$ ± SD—mean ± standard deviation, HD—Huntington’s disease.

**Table 3:** Correlations between plasma levels of BDNF and neurological assessment determined using Spearman’s test.

Correlations/HD Stages	All HD		Early		Intermediate		Advanced	
	R	*p*	R	*p*	R	*p*	R	*p*
BDNF and vertical pursuit	n.s.	n.s.	n.s.	n.s.	n.s.	n.s.	0.94	0.01
BDNF and horizontal pursuit	n.s.	n.s.	n.s.	n.s.	n.s.	n.s.	0.94	0.01
BDNF and speed of horizontal saccades	n.s.	n.s.	n.s.	n.s.	n.s.	n.s.	0.94	0.01
BDNF and dysarthria	n.s.	n.s.	n.s.	n.s.	n.s.	n.s.	0.89	0.04
BDNF and bradykinesia	−0.35	0.02	−0.49	0.04	n.s.	n.s.	n.s.	n.s.
BDNF and torso dystonia	n.s.	n.s.	0.51	0.04	n.s.	n.s.	n.s.	n.s.
BDNF and right upper limb dystonia	n.s.	n.s.	n.s.	n.s.	n.s.	n.s.	−0.97	<0.01
BDNF and left upper limb dystonia	n.s.	n.s.	n.s.	n.s.	n.s.	n.s.	−0.97	<0.01
BDNF and left lower limb dystonia	n.s.	n.s.	0.62	0.02	n.s.	n.s.	−0.97	<0.01
BDNF and motor UHDRS	n.s.	n.s.	n.s.	n.s.	n.s.	n.s.	−0.85	0.04

R—Spearman’s correlation coefficient, n.s.—not statistically significant.

**Table 4:** Correlations between plasma levels of BDNF and neuropsychological assessment determined using Spearman’s test.

Correlations/HD Stages	All HD		Early		Intermediate		Advanced	
	R	*p*	R	*p*	R	*p*	R	*p*
BDNF and the MMSE test	n.s	n.s	n.s.	n.s.	n.s.	n.s.	n.s.	n.s.
BDNF and MoCA	n.s	n.s	n.s.	n.s.	n.s.	n.s.	n.s.	n.s.
BDNF and clock drawing test	n.s	n.s	n.s.	n.s.	n.s.	n.s.	n.s.	n.s.
BDNF and Beck’s scale	n.s	n.s	n.s.	n.s.	n.s.	n.s.	n.s.	n.s.
BDNF and functional assessment	n.s	n.s	0.58	0.03	n.s.	n.s.	n.s.	n.s.
BDNF and the independence scale	n.s	n.s	0.69	< 0.01	n.s.	n.s.	n.s.	n.s.

R—Spearman’s correlation coefficient, n.s.—not statistically significant, MMSE—Mini-Mental State Examination, MoCA–Montreal Cognitive Assessment.

**Table 5:** Correlations between plasma levels of BDNF and cognitive functions determined using Spearman’s test.

Correlations/HD Stages	All HD		Early		Intermediate		Advanced	
	R	*p*	R	*p*	R	*p*	R	*p*
BDNF and VF Repetitions 0–60 s	−0.27	0.04	n.s.	n.s.	−0.41	0.04	n.s.	n.s.
BDNF and TF III Correct words	n.s.	n.s.	n.s.	n.s.	n.s.	n.s.	−0.86	0.04
BDNF and SCNT Correct answers	n.s.	n.s.	0.53	0.04	n.s.	n.s.	n.s.	n.s.
BDNF and SWRT Correct answers	n.s.	n.s.	n.s.	n.s.	n.s.	n.s.	−0.87	0.04
BDNF SWRT Incorrect answers–corrected	0.31	0.04	n.s.	n.s.	0.41	0.04	n.s.	n.s.
BDNF and SIT Correct answers	n.s.	n.s.	0.56	0.04	n.s.	n.s.	n.s.	n.s.
BDNF and TMT part 1 Incorrect answers	n.s.	n.s.	n.s.	n.s.	−0.4	0.04	n.s.	n.s.
BDNF and TMT part 2 Filling time	n.s.	n.s.	−0.53	0.04	n.s.	n.s.	n.s.	n.s.
BDNF and TMT part 2 Correct answers	n.s.	n.s.	n.s.	n.s.	n.s.	n.s.	−0.94	0.04

R—Spearman’s correlation coefficient, n.s.—not statistically significant, VF—verbal fluency, TF—phonemic fluency, SCNT—Stroop color naming test, SWR—Stroop word reading, SIT—Stroop interfence test, TMT—trail making test.

## Data Availability

Data are contained within the article.
